# Whole-genome cartography of p53 response elements ranked on transactivation potential

**DOI:** 10.1186/s12864-015-1643-9

**Published:** 2015-06-17

**Authors:** Toma Tebaldi, Sara Zaccara, Federica Alessandrini, Alessandra Bisio, Yari Ciribilli, Alberto Inga

**Affiliations:** Centre for Integrative Biology (CIBIO), University of Trento, via delle Regole 101, 38123 Mattarello, TN Italy

**Keywords:** p53, Response element, Transactivation potential, Distal enhancer

## Abstract

**Background:**

Many recent studies using ChIP-seq approaches cross-referenced to trascriptome data and also to potentially unbiased *in vitro* DNA binding selection experiments are detailing with increasing precision the p53-directed gene regulatory network that, nevertheless, is still expanding. However, most experiments have been conducted in established cell lines subjected to specific p53-inducing stimuli, both factors potentially biasing the results.

**Results:**

We developed *p53retriever*, a pattern search algorithm that maps p53 response elements (REs) and ranks them according to predicted transactivation potentials in five classes. Besides canonical, full site REs, we developed specific pattern searches for non-canonical half sites and 3/4 sites and show that they can mediate p53-dependent responsiveness of associated coding sequences. Using ENCODE data, we also mapped p53 REs in about 44,000 distant enhancers and identified a 16-fold enrichment for high activity REs within those sites in the comparison with genomic regions near transcriptional start sites (TSS). Predictions from our pattern search were cross-referenced to ChIP-seq, ChIP-exo, expression, and various literature data sources. Based on the mapping of predicted functional REs near TSS, we examined expression changes of thirteen genes as a function of different p53-inducing conditions, providing further evidence for PDE2A, GAS6, E2F7, APOBEC3H, KCTD1, TRIM32, DICER, HRAS, KITLG and TGFA p53-dependent regulation, while MAP2K3, DNAJA1 and potentially YAP1 were identified as new direct p53 target genes.

**Conclusions:**

We provide a comprehensive annotation of canonical and non-canonical p53 REs in the human genome, ranked on predicted transactivation potential. We also establish or corroborate direct p53 transcriptional control of thirteen genes. The entire list of identified and functionally classified p53 REs near all UCSC-annotated genes and within ENCODE mapped enhancer elements is provided. Our approach is distinct from, and complementary to, existing methods designed to identify p53 response elements. *p53retriever* is available as an R package at: http://tomateba.github.io/p53retriever.

**Electronic supplementary material:**

The online version of this article (doi:10.1186/s12864-015-1643-9) contains supplementary material, which is available to authorized users.

## Background

The p53 tumor suppressor is certainly one of the most studied sequence-specific transcription factor to date. Yet, much has still to be learned to fully describe its transcriptional regulatory network, both in terms of the crosstalk with other transcription factors and in terms of the entire spectrum of regulated transcriptional target genes, that can be both up-regulated or down-regulated [1–6].

Recently, several genome-scale techniques such as ChIP-on-chip, ChIP-seq, and, more recently, ChIP-exo, have provided us with different and largely non-overlapping maps of p53 bound sites in the human genome in response to specific stimuli [7–17]. Correlation between occupancy data and modulation of transcription levels of nearby genes helped identifying additional direct p53 target genes, of which >200 have been established [2, 15]. Furthermore, new methodologies are refining the potential to map the p53 network taking also into account the kinetics of transcriptional initiation [18, 19]. It is worth noting that, to date, most experiments have been developed in cancer-derived cell lines that may represent an adapted environment potentially biasing a comprehensive annotation of physiological p53 target sites [7, 20]. To this respect, the impact of specific p53-inducing stimuli and the differentiation/tissue context of the cell have not been systematically investigated [4, 7, 8, 21, 22].

Considerable attention has been given to the sequence and structural features of p53 binding sites that provide for p53 recruitment to target sites [2, 5, 6]. It is now more clear that the loose definition of p53 response element (RE) [23] that has been used for many years comprises a wide range of DNA binding affinity, occupancy rates and transactivation potentials measured by various types of assays, and that specific differences in the definition of p53 REs are evident between purely *in vitro* biochemical assays and *in vivo* occupancy measurements [24–28].

The canonical p53 consensus found in many identified binding sites of mostly up-regulated p53 target genes consists of two copies of the palindromic half-site RRRCWWGYYY separated by a spacer of 0–13 bp, in which R = purine, W = A or T and Y = pyrimidine. Theoretically, each p53 monomer binds five nucleotides – i.e., one monomer binds the I° quarter site R_1_R_2_R_3_C_1_W_1_ and the second monomer the II° quarter site W_2_G_1_Y_1_Y_2_Y_3_-. As reviewed previously, the rather degenerate p53 consensus sequence, reflects the established observation that in virtually all cases of validated p53 REs, an optimal consensus site is not found, because of mismatches, in some cases resulting in partial binding sites, referred to as non-canonical REs [5, 24, 29]. This has raised the hypothesis of a selection pressure to limit the intrinsic potential of p53 proteins to target binding sites, thereby allowing for modulation of p53-induced transcriptional changes by signal transduction pathways affecting p53 protein amount, DNA binding potential, quaternary structures and/or availability of multiple trans-factors [30–36]. For example, p53 REs with lower DNA binding affinity appear to be more frequent in target genes involved in apoptosis [28]. Consistent with this hypothesis, optimized p53 REs have been recently studied in experimental models and *in vitro* for their kinetic and thermodynamic interactions with p53 as well as transactivation potential and shown to provide for high level of p53-mediated transactivation even at low p53 protein levels [25].

Functional assays in a defined experimental setting provided by the yeast *S. cerevisiae* have been extensively used to characterize the transactivation potential of p53 RE in isogenic conditions and exploit variable expression of p53 under an inducible promoter to yield a matrix of transactivation results, to some extent comparable in precision to that of a biochemical assay in a test tube [5, 24, 26, 28, 37–41]. Further, high correlation was reported between results in yeast and transactivation or occupancy data in cancer cell lines [24, 27]. For example, experiments in this model system led to identify functionally active half-site and 3/4 site (3Q) p53 REs, a group of REs collectively considered as non-canonical that were then mapped and validated also in human cells [7].

Here we have combined all the data obtained so far with the yeast-based p53 transactivation assay and developed an algorithm, p53retriever, to scan DNA sequences, identify p53 REs and classify them based on predicted transactivation potential into five broad categories. As unique features, this algorithm takes into account cooperative interactions between groups of mismatches in two p53 dimers and scores also non-canonical REs.

Specifically we used this approach to map functional p53 REs in the proximity of all annotated coding genes, searched for high affinity p53 REs in the entire genome, and mapped functional p53 REs within ENCODE-defined distant enhancer regions. The predictive power of mapping p53 REs with high functional score near transcription start sites (TSS) was validated for a panel of 13 genes, using cell lines differing for p53 status, two p53-inducing stimuli and measuring relative expression by qPCR at three time points. APOBEC3H, E2F7, GAS6, TRIM32, PDE2A, KCTD1, DICER, MAP2K3, DNAJA1, HRAS, KITLG, TGFA and potentially YAP1 were confirmed or identified as p53 target genes.

## Results and discussion

### Development and implementation of p53retriever, a pattern search code that identifies canonical and non-canonical p53 REs based on predictions from transactivation assays

In general, the degree of p53 binding depends on various factors including the state of the p53 protein, its cofactors, and the sequence composition of the p53-RE [5, 32]. Because easier to predict than the p53 state, computational algorithms were developed to explore p53 binding through sequence motif analysis. The majority of these algorithms, such as p53MH [42], do not directly consider the response element (RE) potential to drive p53-dependent transactivation. On the contrary, p53retriever is based on a set of manually curated rules, derived from a compendium of p53 transactivation data obtained using a yeast-based assay [24, 26, 37, 43, 44].

**Fig. 1:**
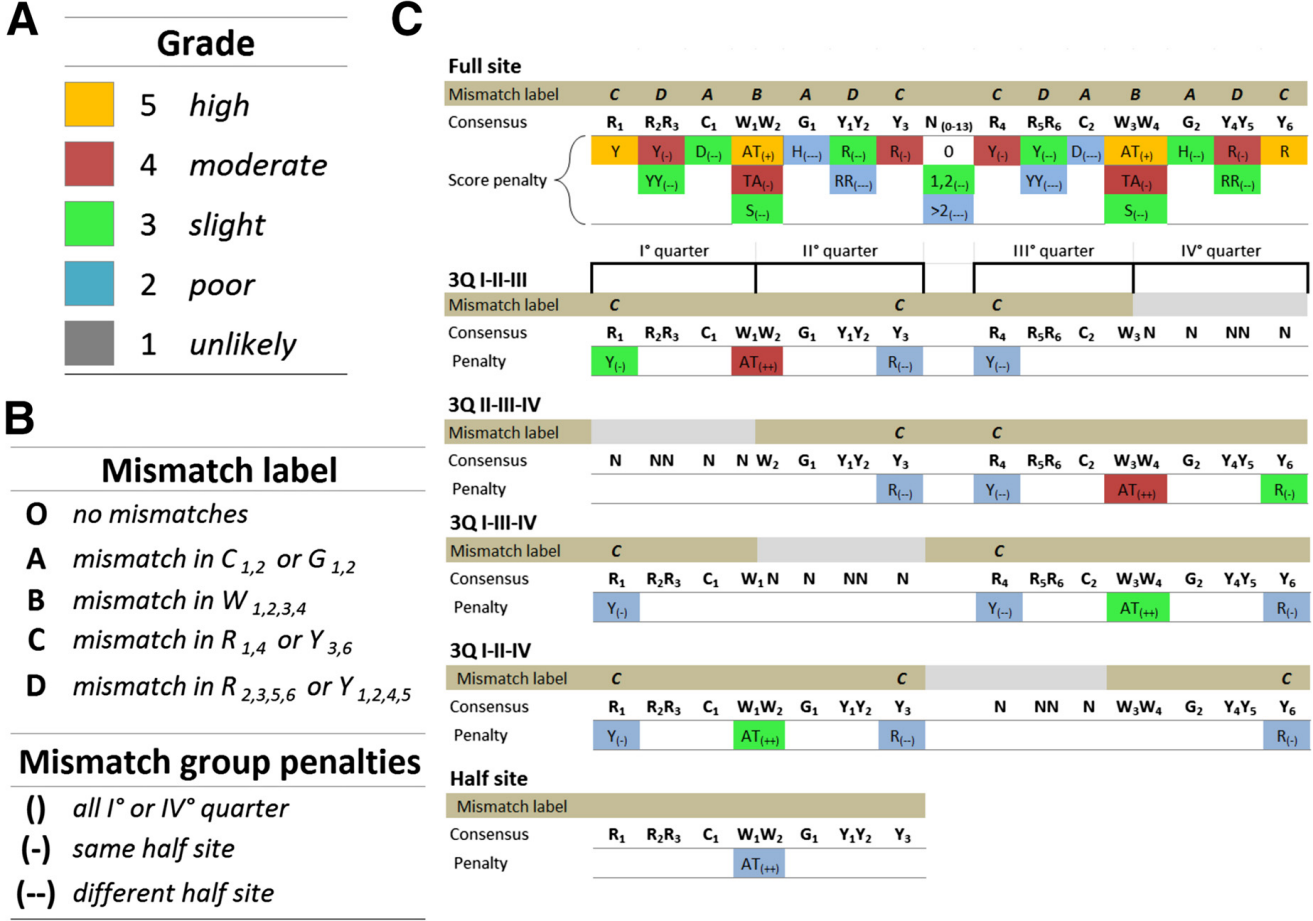
Summary of RE sequence features and associated grades in p53retriever. **a** Grade: classification of REs reflecting associated functional scores. The color code matching the 5 different grades will be maintained in all figures. **b** Mismatch label: classification of mismatches in different positions. High penalties are given to mismatches located in the core consensus sequence (label A and B), lower penalties are given when mismatches are gradually distant from the core (label C and D). Label O is given to a site without mismatches. Mismatches group penalties: different penalties are attributed to groups of mismatches according to how they are scattered or grouped along the site. **c** Schematic representation of the main rules on which p53retriever search algorithm is based. The full list of rules is listed in Additional file 1. The p53 consensus sequence is presented, grouping dinucleotide motifs that were revealed to provide a specific impact on transactivation potentials, based on our previous studies (see text for details) [28]. Penalties are indicated by an increment in the number of the “-” symbol and a color code broadly matching the grade scale. Single mismatches are more penalized when affecting a base in the internal portion of the RE, as indicated. On the contrary, the AT motif at the center of the CWWG core is a positive feature, particularly in the case of non-canonical REs (3Q = 3Q sites and half sites)

REs are scored from five (= highly functional REs activity) to one (= unlikely functional REs) (Fig. 1a). The grade represents the inferred transactivation potential rather than being an indication of the percent similarity to the canonical p53 consensus sequence. For full site p53 REs the grade considers a severe negative impact of a spacer between the two half sites larger than two nucleotides (Fig. 1c). Variable p53-RE spacer lengths are known to affect transactivation capacity. Only two previous studies tried to incorporate the spacer length as one of the relevant features [11, 45], calculating a penalty score directly proportional to spacer length. Also in our algorithm, based on previous results, we attribute high negative impact to spacers longer than two nucleotides (Fig. 1c). Indeed, REs with a long spacer length are also confirmed to be rarely bound by p53 *in vivo* [7, 14, 46, 47]. Many of the computational approaches for identifying putative p53-REs define how similar that putative binding site is to the consensus, but do not consider the local context of single mismatches within the RE. In our approach mismatches from consensus are also weighted depending on their position within the RE 20-mer sequence, given the finding that mismatches in the quarter sites at the interface between the two half sites have a more severe impact likely due to cooperative interactions among two p53 dimers [28] (Fig. 1b). In addition, interaction effects between groups of mismatches are also considered. In general, any combination of mismatches is penalized in a different way according to their location, considering that p53 is functionally active as a tetramer, that each p53 monomer interacts with a 5 nt motif (quarter site) and that the p53 tetramer is thought to be assembled as a dimer of dimers [48]. If groups of mismatches are localized in the same “quarter” of the RE, the score is less penalized than if the same mismatches were scattered in different quarters (Fig. 1b). Importantly, non-canonical REs consisting of 3Q sites and ½ sites [5, 7] are considered functional p53 REs with specific pattern searches. A graphical view of these features presented as “penalty matrix” summarizes the main features of our pattern search (Fig. 1c). The complete list of the rules used to attribute the functional score is presented (Additional file 1). The p53retriever pattern search algorithm, together with functions to better visualize search results, has been implemented as an R package and is available for download at: http://tomateba.github.io/p53retriever.

### Distribution of identified p53 response elements around human promoters

We applied p53retriever to the set of sequences in the human genome placed around annotated transcriptional start sites (TSS), selecting a window from -10 kb to 10 kb. The entire list of identified REs, chromosomal coordinates, official gene name, distance from TSS and RE sequence features resulting in the given grade, is available in Additional file 2.

**Fig. 2:**
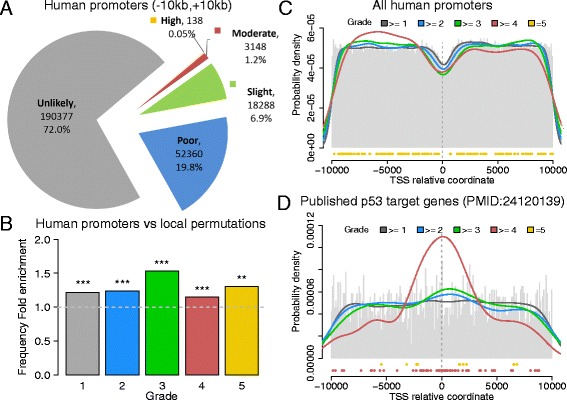
Analysis of p53 REs found in human promoters. **a** Pie chart displaying the distribution of grades associated to REs found in all human promoters. **b** Comparison between the frequencies of REs found in human promoters, and the frequencies of REs found in scrambled promoter sequences after applying local permutations (to preserve the local GC content). The comparison is shown for each grade. The ratio is 1 if the frequency is the same, > 1 if the frequency is higher in real promoters, <1 if the frequency is higher in scrambled promoters. All enrichments are significant, the binomial test p-value is 4.84E-04 for grade 5 (**), <1E-15 for all the other grades (***). **c** p53 occupancy metaprofile, based on the position of REs in all human promoters, centered on the TSS position. The grey histogram displays the probability distribution of all REs independently from the grade. Colored lines represent the density distribution of REs with higher grades (the grade threshold corresponding to each color is displayed in the legend). The specific positions of grade 5 REs are dotted in yellow under the histogram. **d** p53 occupancy metaprofile, based on the position of REs in the human promoters of 228 p53 target genes, published in [15]. The color scheme is the same as in panel C. The specific positions of grade 4 and 5 REs are dotted under the histogram (in yellow for grade 5, red for grade 4)

The distribution of identified p53 REs grouped based on the functional score, shows a very large preponderance of “grade 1” REs, that are considered as unlikely functional (Fig. 2a). Also, the distribution of RE scores is highly skewed, with only 0.05 % of REs obtaining the highest grade, supporting the hypothesis of a selecting pressure to reduce p53 binding affinity and provide plasticity in the modulation of p53-mediated stress responses *in vivo* [4, 28]. Very recent analyses confirmed that p53 REs that are more highly conserved in evolution are relatively weak p53 RE sites displaying lower levels of occupancy compared to higher affinity REs that exhibit low evolutionary conservation [47]. Grade five sequences either lack entirely mismatches, or contain two or fewer mismatches in the external positions (R1,Y6/R4,Y3, see Fig. 1c), and contain the positive AT motif in the CWWG core. The vast majority of REs that can be considered functional are in the grade two category. Predicted to be poorly responsive on their own, these REs could participate in the regulation of gene expression conditional to other features, such as the local sequence context of promoter architecture. Included in the grade two category are ~30 % of all half sites mapped (Fig. 2a). A unique feature of our search tool is the specific pattern search for non-canonical 3Q sites. Interestingly, even though mismatches in the two internal quarter sites have an higher impact on p53 transactivation for 3Q sites compared to full sites, and thus result in a final lower grade, many 3Q sites obtained a grade higher than 2. Hence, a great number (13,744) of p53 REs are predicted to be functional even though the entire motif is not present. This observation strongly supports recent reports suggesting that p53 REs match the consensus in one half site, with the two central quarter sites being somehow less variable [14]. It is also consistent with the recent report of the frequent identification of p53 half-sites among p53 ChIP-seq peaks lacking full sites [47].

We compared the results obtained searching within human promoters with what we would expect by chance, by applying p53retriever to sets of scrambled sequences obtained by local permutations of real promoter sequences (see Methods and Additional file 3: Table S1). Local permutations allowed us to preserve the local GC content of promoter regions, showing in fact an increase in GC content around the TSS (see Additional file 3: Figure S1). From this analysis we could determine that the frequency of REs in the global set of human promoters is slightly but significantly higher than the frequency of REs in scrambled sequences (Fig. 2b). This soft enrichment is plausible, given that we are considering all known human TSS and not specific populations of genes. Grade five and three are the most enriched class of REs when comparing the frequency of each grade (Fig. 2b).

Mapping all the REs considering their position with respect to the TSS, we obtained an occupancy metaprofile of p53 REs, displayed in Fig. 2c. This occupancy profile reveals a general decrease of REs in the region proximal to TSS (from -2 kb to +2 kb). This decrease affects all REs, independently from the grade, and appears to be a consequence of the local increase in GC content, since we observed the same effect in scrambled sequences when applying local permutations (Additional file 3: Figure S2). Overall the REs reduction (approximately of ¼.) could be interpreted as a selection against a high density of active p53 REs from promoter regions of non-target genes that is limited to about 2 kbs from TSS. This reduction is driven by the general increase in GC content around the TSS, which more globally is instrumental in the interplay between chromatin conformation and transcription processes. On the other hand, when restricting our analysis to the promoter region of known p53 targets, we found an entirely different landscape. Fig. 2d displays the promoter occupancy metaprofile of REs identified by p53retriever in a group of 189 HGNC genes listed as targets of p53 in literature and collected in [15, 45]. Interestingly, this profile shows the highest probability density in the region closer to the TSS, especially for functional REs with grade four and five (red line in Fig. 2d). Indeed, recent data reported a prevalence of p53 REs nearby the TSS of known target genes [16, 47].

### Comparison with other p53 binding site datasets and search tools

To further verify if p53retriever recognized already established p53 binding sites, we compared our approach with lists of p53 target genes and REs previously reported. The detailed results of all comparisons are contained in Additional file 4.

**Fig. 3:**
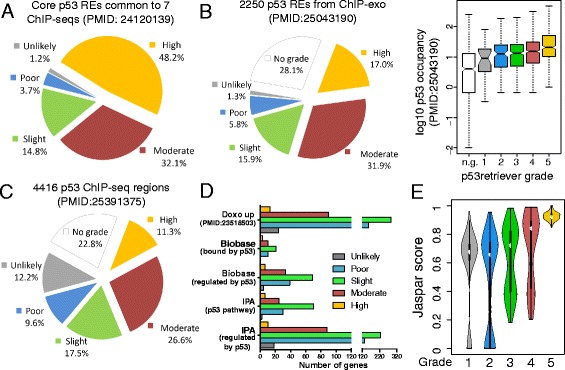
Comparison of p53retriever with other p53 binding site datasets and tools. **a** Pie chart displaying p53retriever classification on a list of 81 regions commonly identified by 7 different ChIP-seq experiments [15]. **b** Left panel: pie chart displaying p53retriever classification on a list of 2250 regions identified by ChIP-exo [14]. Right panel: boxplot displaying for each grade assigned by p53retriever to ChIP-exo sequences, the distribution of the corresponding ChIP-exo occupancies, measured in [14]. n.g. = no grade given by p53retriever. **c** Pie chart displaying p53retriever classification on a list of 4416 regions identified by ChIP-seq in [17]. **d** p53 target gene lists from curated databases (Biobase and IPA) or from expression datasets (Doxo up: genes up-regulated upon doxorubicin treatment) were compared to the list of p53 promoter REs obtained with p53retriever. Presented in the bar graph are the predicted p53 REs grouped by the maximum functional grade identified by p53retriever in their promoter. **e** Comparison between p53 REs identified in human promoters by p53retriever, and the corresponding score given by Jaspar p53 PWM (MA0106.2), based on ChIPseq data. REs are divided in 5 groups along the horizontal axis, corresponding to the grade assigned by p53retriever. For each group, the distribution of the scores given by Jaspar PWM is represented as a violin plot, i.e., a box plot with a rotated kernel density plot on each side. Jaspar scores range from 0 (the RE is not identified) to 1 (the RE is optimal)

First, we used our method to score 81 REs sequences that are consistently bound by p53 according to seven different ChIP-seq datasets, reported in [15]. All these sequences were picked by p53retriever as potentially functional. Interestingly, excluding one sequence, all p53 REs from this list obtained a grade greater than one with the majority being of grade 5, confirming that our tool can discriminate functional and well-known REs (Fig. 3a).

Next, we applied p53retriever on p53 REs obtained by Chip-exo analysis [14], providing near-nucleotide resolution of p53 bound sites in response to a variety of genotoxic stresses. (Fig. 3b). While 28 % of sites were not classified, the majority of bound sequences from ChIP-exo obtained a grade greater than 1, with a predominance of grade four and five, (Fig. 3b, left panel). Interestingly, we saw a clear correlation between higher relative occupancy and higher RE grade (Fig. 3b, right panel). Looking in more detail to the “no grade” group, we noticed that all non-scored sequences differed from the canonical RE site for features which are highly penalized by our algorithm, like a number of mismatches higher than three scattered on three different quarter sites. Nevertheless, we could show that these not-scored sequences are mostly characterized by low occupancy values (white boxplot in Fig. 3b right panel, Wilcoxon rank sum test *p*-value=1.29E-09). Consistently, when considering the subset of regions increasingly bound by p53 after all the stimuli used in [14], the percentage of “no grade” drops to 17.6 % (Additional file 3: Figure S3).

We also extended the comparison to a Chip-seq dataset, reported in [17] (Fig. 3c) and obtained an overall similar distribution of RE grades. The percentage of regions with “no grade” is 22.8 %.

Next, we extended the comparisons to other lists of REs, starting from two small collections of reported p53 REs, based on heterogeneous experimental approaches [2, 15, 45]. Only a minority of those REs obtained the highest grade, and the proportion of sequences not scored as potentially functional was approximately 40 % (Additional file 3: Figure S4). It has to be said that the REs reported in those lists are not guaranteed to be the ones actually or solely responsible for the responsiveness of the associated genes to p53.

Even though total mRNA levels are an indirect measurement of p53 transcriptional activity, they reflect the transcriptome status upon p53 activation. Thus, we did an additional comparison using microarray data obtained after p53 activation upon Doxorubicin treatment of MCF7 cells [49]. The majority of differentially expressed, up-regulated genes turned out to have a p53 binding sites with grade three (Fig. 3d), and exhibited a specific enrichment of REs with grade >3 near the TSS (Additional file 3: Figure S5). Similar comparisons were done with lists of p53 target genes in curated databases such as TRANSFAC and IPA. Again, the majority of these genes have a RE of grade three predicted by p53retriever in their promoter region (Fig. 3d). Using Ingenuity Pathway analysis (IPA), grade five and grade four human promoters revealed a strong p53 pathway signature (Additional file 5).

Finally, we compared p53retriever results with the standard PWM approach, using two PWMs provided by the JASPAR database (see Methods). All REs identified by p53retriever in the set of human promoters were scored in parallel with both JASPAR PWMs: the comparison with the JASPAR PWM derived from ChIPseq data is shown in Fig. 3e. Although there is a high agreement on REs with the maximum grade, very close to the optimal p53 consensus, the comparison shows divergences between the two methods for the lower grades. For example, a considerable population of REs assigned to grade four by p53retriever receives very low scores from JASPAR. This is likely due to the presence of ¾ sites that are over-penalized by the PWM approach. On the other hand, many REs with low grades are highly scored by JASPAR, that doesn’t penalize groups of scattered mismatches. Apart from grade 1, we can observe a linear trend between the two scoring systems if we look at the median values of the boxplots displayed in Fig. 3e, so we can conclude that the two approaches are distinct and complementary. On the other hand, the second JASPAR matrix, based on SELEX data, gives misleading results, since even optimal REs (grade 5) receive low scores (Additional file 3: Figure S6).

### High grade p53 REs are enriched in distant enhancers

**Fig. 4:**
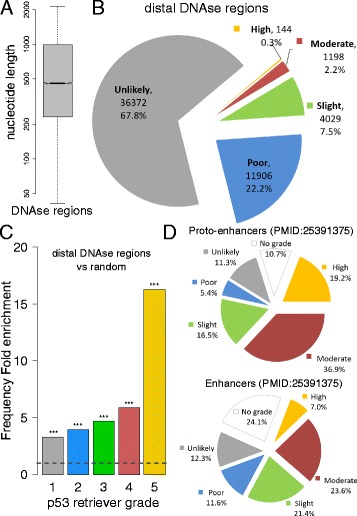
Analysis of p53 REs found in distal DNAse hypersensitive regions. **a** Boxplot of the nucleotide length of human distal DNAse hypersensitive regions, determined as described in Methods. **b** Pie chart displaying the distribution of grades associated to REs found in human distal DNAse hypersensitive regions. **c** Comparison between the frequencies of REs found in human distal DNAse hypersensitive regions, and the frequencies of REs found in random sequences with the same GC content. The comparison is shown for each grade. The ratio is 1 if the frequency is the same (horizontal dotted line), > 1 if the frequency is higher in DNAse regions, <1 if the frequency is higher in random sequences. All enrichments of REs mapped to distal DNAse hypersensitive regions are highly significant (Binomial test p-value < 1E-20). **d** Pie charts displaying p53retriever classification on two lists of regions identified by ChIP-seq in [17] as proto-enhancers (upper panel) and enhancers (lower panel)

Recent functional genomics approaches, particularly resulting from the ENCODE initiative, have revealed that transcription is rather pervasive, that enhancer sequence can be very distant, at least in terms of primary sequence, from genes, and that active enhancers can be mapped based on specific histone code marks [50, 51]. Hence, we exploited this rich body of available information to map p53 REs in distal enhancer sites, using DNAse hypersensitive sites tracks. We filtered out sites overlapping with promoter regions defined in the previous sections, and considered a population of 43,787 distal regions, whose length distribution is displayed in Fig. 4a. p53retriever was run on this set of regions, and the complete results are provided in Additional file 6. The grade distribution of REs found in distal DNAse regions is displayed in Fig. 4b. The frequency of REs in these regions is significantly higher than the frequency found in human promoters and also in random sequences (Fig. 4c and Additional file 3: Figure S7). The overall fold enrichment is 3.54, but this trend grows proportionally to the grade of the REs, reaching a peak with grade 5. In fact, 144 high grade REs are found within DNAse hypersensitive sites (Fig. 4b), more than in the entire human promoter dataset. The fold enrichment of grade five REs is 16.3 (Fig. 4c). Presently, it is undetermined if this enrichment for high quality binding sites reflects a common trend for sequence-specific transcription factors or a distinct feature of p53 family proteins. Consistent with our results, higher levels of p53 occupancy in distal enhancers compared to promoters was very recently reported based on ChIP-seq analysis of lymphoblastoid cell lines treated with doxorubicin [47]. Additionally, Chip-seq analysis reported in [17] allowed us to expand the study of p53 REs in enhancers regions. In fact that paper provided p53 bound regions classified as enhancers based on ENCODE annotation or as proto-enhancers, where p53 could act as pioneer transcription factor. Interestingly, this latter group showed an enrichment for high scoring (grade four and grade five) p53 REs according to p53retriever and a lower proportion of sequences with no grade (Fig. 4d, top panel).

### New direct p53 target genes identified based on the p53 RE functional search tool

**Fig 5:**
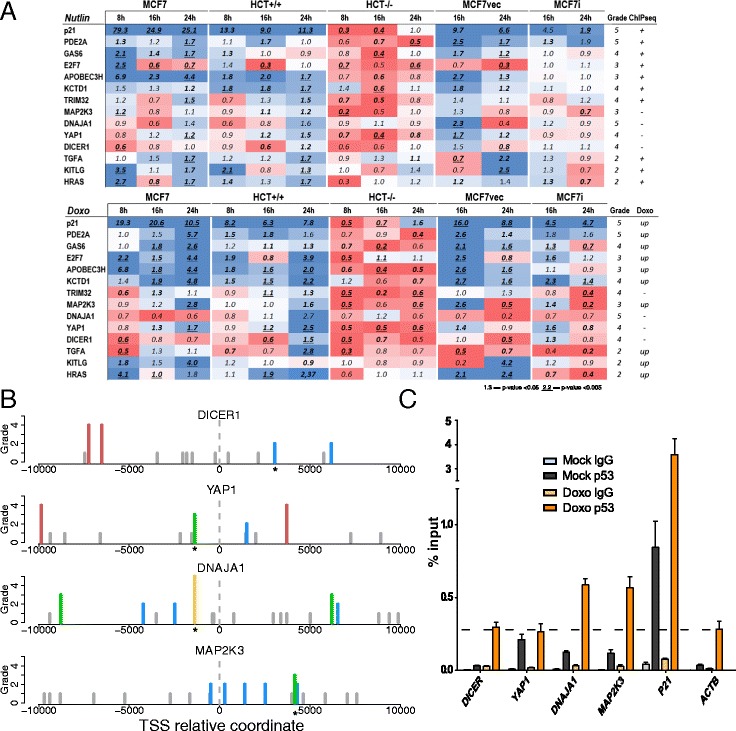
p53-responsiveness of target genes associated with predicted functional REs. **a** qPCR results of 13 selected genes. Fold of changes values (∆∆Cq) upon each treatment are presented as a heatmap. Upper part shows mRNAs level after Nutlin-3A treatment while the lower shows the same after Doxorubicin (Doxo) treatment. Expression levels were tested in different cell lines (MCF7, HCT +/+, HCT −/−, MCF7 vector and MCF7 shp53) and at different time points (8, 16 and 24 h) (Details are presented in Additional file 7). For each gene, the “Grade” column presents the binding site grade reported by our algorithm. “ChIPseq” column shows the presence (+) or absence (−) of reported p53 binding sites [8]. “Doxo” column presents mRNA levels obtained by microarray data [49]. **b** Positional representation of p53 binding sites in TSS proximity for DICER, YAP1, DNAJA1, MAP2K3. Bar height is proportional to the binding site grade. Grade 5 is yellow, grade 4 is red, grade 3 is green, grade 2 is blue and grade 1 is grey. **c** p53 occupancy on the promoter of DICER, YAP1, DNAJA1 and MAP2K3 comparing Mock and Doxo condition in MCF7 cells. P21, well-known p53 direct target, is a positive control. ACTB is used as a reference. Sites validated by ChIP are indicated by a * symbol in Figure 5b

High-activity, or non-canonical p53 REs predicted to be moderately active were mapped by our tools near the TSS of genes that are not completely established or novel putative direct p53 target genes. To infer the predictive power of the pattern search on p53-dependent transcriptional changes, 13 genes were selected and their expression was tested followed by qPCR in cell lines differing for p53 status (MCF7, two derivative clone so called MCF7 vector and MCF7shp53, HCT p53^+/+^ and HCT p53^−/−^) and at different time points (8, 16, 24 h) after p53 activation by two different treatments, i.e., Doxorubicin -a genotoxic chemotherapeutic drug- and Nutlin-3A -an MDM2 inhibitor- (Fig. 5a) (Additional file 7). Results support p53-dependent up-regulation for most genes. The p53-dependency is confirmed by the absence of induction in HCT p53^−/−^ and MCF7shp53 cell lines, despite the different p53 status between the two cells lines (a p53-null and a partial knockdown cell line, respectively). In some cases, the increase in gene expression compared to the mock condition was time-dependent. Differences in these kinetic features were apparent between the two treatments applied. E2F7 was inducible by Doxorubicin at different time points, while after Nutlin-3A treatment an early up-regulation was followed by repression, which appeared to be p53-dependent. GAS6 and KCTD1 had a similar trend especially in MCF7 cells. Differences were noted between MCF7 and the MCF7-vector derivative clone in the magnitude or the kinetics of relative expression changes (*e.g.,* PDE2A, APOBEC3H, KCTD1, DNAJA1, DICER). Nine of the thirteen candidates (PDE2A, GAS6, E2F7, APOBEC3H, KCTD1, TRIM32, TGFA, KITLG, HRAS) were selected among the list of genes having both a predicted binding sites in our algorithm output with a grade higher or equal to 2, and a reported p53 binding sites on ChIPseq datasets [7, 8]. For all of them except TRIM32, total mRNA levels are also reported as upregulated after Doxorubicin treatment by microarray data [49]. Although the induction is not directly proportional to the grade, we confirmed p53 dependent induction by qPCR for all of them in time/cell line dependent manner. Even though TRIM32 is not upregulated after Doxorubicin treatment in all the tested cell lines, it is upregulated upon Nutlin-3A treatment, confirming ChIP-seq data. Besides their p53 binding sites, these candidates were selected because of their reported involvement in cell-cycle control and tumor progression (PDE2A, E2F7, GAS6, TRIM32, HRAS, KITLG and TGFA), in transcription (KCTD1), and DNA editing (APOBEC3H) (see Supplementary Text in Additional file 3).

For the remaining four genes, whose REs are displayed in Fig. 5b, we performed a chromatin-immunoprecipitation experiment in MCF7 cells treated with Doxorubicin for 16 h. Weak p53 occupancy was observed by qPCR at DNAJA1 and MAP2K3 loci after doxorubicin treatment, while a region containing a predicted grade 3 category p53 RE in the YAP1 gene showed evidence for p53 occupancy in the mock condition. Our results did not support direct p53 binding to the DICER promoter, consistent with a previous study [52] (Fig. 5c).

Overall, we propose DNAJA1, MAP2K3 and potentially YAP1 as new direct p53 target genes, although the level of transactivation was relatively low.

DNAJA1 can act as a co-chaperone of Hsc70 that was previously associated to radioresistance phenotype in wild type p53 glioblastoma cells treated with farnesyltransferase inhibitors [53]. Recently, overexpression of DNAJA1 was associated with a reduction of pancreatic cancer cell survival and with c-Jun repression [54].

MAP2K3 participates in the MAP kinase cascade and can phosphorylate p38. This protein was identified as a senescence-promoting factor in human breast epithelial cells [55]. However, it has also been associated to tumor invasion potential and to be regulated at transcriptional level by NFY, NF-κB and gain-of-function mutant p53 [56].

The Yes-associated protein 1 (YAP1) is a transcriptional regulator involved in the Hippo signaling pathway. Evidences support both an oncogenic and a tumor suppressor role for YAP1, linked to ABL1-induced apoptosis [57]. YAP1 protein was found capable to bind the p53 promoter and a positive feedback loop was proposed based on the finding that p53 can bind the YAP promoter [58]. In part consistent with this view we found p53-dependent YAP1 gene up-regulation both after doxorubicin and Nutlin-3A treatment.

## Conclusions

Several previous tools were developed to identify bona fide p53 response elements, starting with pioneering *in vitro* selection experiments that led to the initial and still accepted definition of the consensus p53 RE [11, 42, 45, 59, 60]. The majority of these tools were based on position weight matrices derived from results of *in vitro* approaches, namely competitive gel shift assays and SELEX, more recently integrated with results obtained from Chromatin immunoprecipitation experiments. A systematic effort to quantify changes in DNA binding affinity (dissociation constants) using fluorescence anisotropy titration led to the development of a p53 binding site predictor algorithm [60]. This tool was also used to search genome wide for high affinity p53 REs and to map naturally occurring single nucleotide polymorphisms (SNPs) that can impact on the DNA binding affinity of p53. The functional relevance of SNPs within p53 REs has been established in several reports [15, 24, 27, 44, 61].

All position weight matrix approaches assume additive contributions of the individual positions within the RE sequence, and except for [45] and [11], all tools do not specifically weigh the impact of spacers between half site decameric RE motifs in the 0-13 nt range. This spacer length was in fact considered neutral in the initial *in vitro* experiments [23]. However, DNA binding assays where RE sequence are embedded in longer DNA molecules, competitive binding experiments in microfluidics, Chromatin Immunoprecipitation assays, yeast- and mammalian-cell based transactivation assays all indicate that even a single nucleotide spacer between p53 RE half sites can reduce transactivation potential [5, 24, 27, 44]. In fact, when the spacer is longer than 2–3 nt the two decameric half sites no longer show cooperative interactions [24, 28, 62], although when the distance in primary sequence approach one helical pass, transactivation potential appears to increase beyond additivity [24], yet remaining much lower compared to the absence of a spacer. The negative impact of spacer is even more dramatic for TAp73 [62] and TAp63 proteins, but not for ΔNp63 [63], suggesting that the structure as well as the sequence of DNA binding sites can lead to conformational changes in the quaternary tetrameric structure of p53 family proteins, and that intrinsic differences exist in the oligomerization state of these proteins [64].

We have coded in p53retriever sequence and structural features of p53 REs impacting on transactivation potential that were revealed in the past several years using our yeast-based transactivation assay [5, 26, 28, 63, 65, 66]. The resulting algorithm has several distinctive features compared to previous tools, particularly for scoring interactions among groups of mismatches, non-canonical 3Q sites and half sites p53 REs, weighing the impact of consensus mismatches considering their position within the full site RE sequence, i.e., giving higher penalty to mismatches in the two internal quarter sites, and weighs consensus sequence variations within dinucleotide motifs in the core and flanking regions [28] (Fig. 1, Additional file 1). Possible interactions between nearby half site p53 REs or clusters of full site and 3Q sites are currently not considered by our algorithm.

We mapped and ranked functional REs near TSS for all annotated transcripts in UCSC (Additional file 2). Further, we exploited ENCODE data and provide a cartography of ranked p53 REs within distant DNAse hypersensitive sites, considered as distant enhancers (Additional file 6). In these regions we found a significant 16-fold enrichment of high grade REs with respect to the basal frequency expected by chance or observed in promoter regions. An enrichment for high grade REs was also found among proto-enhancer sequences bound by p53 identified by ChIP-seq [17]. It is worth noting that our results represent a projection from all DNAse hypersensitive sites, irrespective of the specific tissues in which they are active. Tissue variability may influence which REs are selectively bound. An additional layer of complexity is represented by the known interplay between different transcription factors. This important aspect is not included in our analysis that is focused on p53 alone.

Although the data on which the algorithm is construed are the outcome of transactivation assays measured from chromatinized promoter-reporter construct, the isogenic nature of the yeast-based functional assays, minimizes most variables potentially impacting on transactivation by p53; at the same time distinct chromatin features of the natural context of the REs’ location *in vivo* may certainly influence the associated gene transcriptional responsiveness to p53. Hence the yeast-based results might be more similar to ChIP-seq and ChIP-exo results, albeit with a more quantitative power.

Undoubtedly different ChIP-seq experiments do not agree with each other and there is limited overlap among the results obtained with different cell lines or using different treatments to activate p53. While global differences in occupancy could be related to differences in accessibility between different tissue-derived cells or to distinct p53 post-translational modifications or cofactors activated by different treatments, it was interesting to find that the list of p53 bound sites that are common to multiple ChIP-experiments were highly enriched for high scoring (grade four and grade five) REs and none of them failed to be classified by our tool (Fig. 3a). Instead, when examining individual ChIP-seq or even, although to a lower extent, ChIP-exo data, 20 % to 30 % of p53 bound fragments did not contain a motif scored by p53retriever. While those sites may represent examples of p53 proteins tethered to DNA by protein interactions, the manual inspection of “no grade” sites from the ChIP-exo datasets showed that the majority of these sites resemble p53 response elements but contain several (three or more) “core” mismatches scattered on three different quarter sites. These multiple mismatched REs are not presently scored by p53retriever, but would probably result in weak responsiveness. Consistently, the majority of no grade ChIP-exo REs showed lower occupancies (Fig. 3b right panel).

Finally, we decided to validate a few of the predictions from the pattern search, particularly for non-canonical 3Q sites using cell lines as a model. 13 genes with mapped functional REs were chosen. Overall, despite our algorithm doesn’t consider the system complexity of transcriptional regulation in living cells and the response variability upon each different p53 stimulus, results support p53-dependent transactivation for the majority of them. Based on the combined qPCR and ChIP results we conclude that DNAJA1, MAP2K3, and potentially YAP1 can be considered new direct p53 target genes, linking p53 to yet additional potential biological outcomes. Furthermore, our data further establish the very recent findings of PDE2A, GAS6, E2F7, APOBEC3H, KCTD1, TRIM32, HRAS, KITLG and TGFA as p53 target genes.

## Methods

### Implementation of pattern search rules in p53retriever

We implemented the set of manually curated rules (Additional file 1) in an R package called p53retriever. p53retriever source and binary files are available on Github, at (http://tomateba.github.io/p53retriever/). p53retriever contains a main function that identifies potential REs. This function needs as input an arbitrary DNA sequence, and returns a table containing information about the identified REs, such as position, sequence, spacer length, mismatch label and grade. The format of the output is similar to Additional file 6. Many functions are also provided in order to graphically display the results. The package is documented with usage examples, and fully integrated with other CRAN and Bioconductor packages. In particular, p53retriever depends only on the previous installation of the Bioconductor Biostrings package.

### Human promoters dataset

Human promoter sequences were extracted from the UCSC database (http://genome.ucsc.edu/) considering, for each transcript with a distinct TSS, the 20 kB region surrounding the transcription binding site (genome build GRCh37/hg19). The final dataset consists of 23,541 promoter sequences, associated to distinct UCSC identifiers and corresponding to 18,355 HGNC genes.

### Human distal DNase regions dataset

Encode DNase-seq regulatory regions (genome build GRCh37/hg19) were obtained from the following cell lines: Gm12878, H1hesc, Helas3, Hepg2, Hmec, Hsmm, Hsmmt, Huvec, K562, Monocd14, Nha, Nhdfad, Nhek, Nhlf, Osteobl, Hsmmfshd, Lncap, Nb4, Nt2d1,Panc1. The consensus was defined as the merge of all the regions that were present in at least two cell lines. Only distal regions, with more than 10 kb from the nearest annotated TSS, were kept in the dataset, in order to avoid overlap with promoter regions. The final dataset consists of 43,787 regions, with a mean length of 673.3 bases.

### Simulations with random sequences

Sets of scrambled promoter sequences were generated by local permutations (bin size = 500 nt) of human promoter sequences (−10 kb, +10 kb from TSS). This allowed to preserve the local GC content in the random model; p53 REs were then identified and classified with p53retriever. The random simulation was run ten times, and the results were compared to REs identified in real human promoters.

Set of random sequences were generated, with the same number and the same GC content (44 %) of human DNA sequences; p53 REs were then identified and classified with p53retriever. The random simulation was run ten times, and the results were compared to REs identified in human distal DNase regions promoters.

### Pathway analysis of DEGs

All pathways analyses were performed using IPA (www.ingenuity.com). Only direct interactions were considered in the setting parameters.

### Comparison with other datasets

Several lists of p53 targets, identified by their HGNC symbol, were extracted from online databases such as Biobase TRANSFAC (http://www.biobase-international.com/product/transcription-factor-binding-sites) and IPA, or from previous publications, referenced in the main text. These lists were used to select populations of genes among our dataset of human promoters, and analyze the grade of the REs identified by p53retriever (as shown in Fig. 2d).

Several sets of p53 RE sequences or p53 bound regions were taken from previous publications, referenced in the main text. p53retriever was run directly on these sequences (as shown in Fig. 3a, b, c).

### Comparison with JASPAR PWMs

Two PWMs for p53 were downloaded from the JASPAR database (http://jaspar.genereg.net/). One PWM, MA0106.1, is built on SELEX data, while the second, MA0106.2, is built on ChIPseq data. The original values of the downloaded PWMs were based on nucleotide frequencies and therefore more similar to Positional Frequency Matrices. These frequency values were transformed in log2 probability ratio values with the PWM function implemented in the Bioconductor Biostring package, using a multinomial model with a Dirichlet conjugate prior to calculate the estimated probability of base b at position i. The final score of a match ranges from 0 to 1. All REs identified by p53retriever in the set of human promoters were scored with JASPAR PWMs: the comparison with MA0106.2 is shown in Fig. 3d, while the comparison with MA0106.1 is shown in Additional file 3: Figure S2.

### Cell lines and culture conditions

The human breast adenocarcinoma-derived MCF7 cell line (p53 wild type) was obtained from the InterLab Cell Line Collection bank, ICLC (Genoa, Italy) while the colon adenocarcinoma HCT116 (p53^+/+^) cell line and its p53^−/−^ derivative were a gift from B. Vogelstein (The Johns Hopkins Kimmel Cancer Center, Baltimore, Maryland, USA). MCF7 cells stably expressing an shRNA targeting p53 (MCF7shp53) or control cells (MCF7vector) were kindly provided by Dr. Agami (Netherlands Cancer Institute, Amsterdam). Cells were normally maintained in DMEM or RPMI (BioWhittaker, Lonza, Milan, Italy) supplemented with 10 % FCS, antibiotics (100 units/ml penicillin plus 100 mg/ml streptomycin) and 2 mM glutamine. Puromycin (Sigma-Aldrich, Milan, Italy) was used to maintain the selection, at 0.5 μg/mL as final concentration.

### RNA extraction

Cells were seeded into 6-well plates and allowed to reach 70-80 % of confluence before treating with 1.5 μM Doxorubicin or 10 Μm Nutlin-3A. Doxorubicin was purchased from Sigma-Aldrich (Milan, Italy) while Nutlin-3A was obtained from Alexis Biochemicals (Enzo Life Science, Exeter, UK). After 8 h, 16 h or 24 h of treatment cells were harvested and total RNA was extracted using RNeasy Mini Kit (Qiagen, Milan, Italy) according to the manufacturer’s instructions. In-column DNAse treatment (RNase-Free DNase Set, Qiagen, Hilden, Germany) was performed to remove DNA contamination during the extraction. Purity of RNAs (A260/A280 value of 1.8–2.1) and concentration were measured using the Nanodrop spectrophotometer.

### qPCR

cDNA was generated starting from 1 μg of RNA by using the RevertAidTM First Strand cDNA Synthesis Kit (Fermentas, Milan, Italy) in 20 μL as final volume following manufacturer’s instructions. Primers were designed by Primer-BLAST performing in silico analysis as well as standard curves to define assay specificity and efficiency (Additional file 7). All qPCR assays were performed on CFX Touch Real-Time PCR Detection System (Bio-rad, Milan, Italy) in a 384-well plate format. Optimal primer concentrations (200nM-400nM) were determined by identifying conditions resulting in the lowest C_q_ combined with absence of primer dimer formation. Reaction volumes were set at 10 μl. SYBR Green assays contained 5X KAPA SYBR FAST qPCR Mastermix (Kapa Biosystems, Resnova, Rome, Italy), 400 nM each primer (MWG, Operon, Ebersberg, Germany) and 25 ng of cDNA. Initial thermal cycling conditions were 1 cycle of 95 °C for 3 mins, followed by 40 cycles of 95 °C for 30 s, 60 °C for 20 s, 72 °C for 60 s. At the end a melt curve analysis was performed. Post-run relative mRNA quantification was obtained using the comparative C_q_ method (ΔΔC_q_), where glyceraldehyde 3-phosphate dehydrogenase (GAPDH) and β-2microglobulin (B2M) served as reference genes.

### ChIP assays

MCF7 cells were cultured in complete medium in a 150-mm Petri dishs and when reaching 70/80 % confluence were treated for 16 h with Doxo. The procedure for crosslinking, sonication, IP and analysis followed a previously described protocol [49]. Antibodies used for ChIP assays were: p53 (DO-1) and IgG (sc-2025 or sc-2027) (Santa Cruz Biotechnology®) (Millipore). ChIP analysis was performed with the comparative Cq method (ΔΔCq) and normalized as % of input, using β-actin gene as negative control and p21 as positive control for p53 enrichment.

### Availability of supporting data

The data sets supporting the results of this article are included within the article and its additional files. p53retriever source and binary files are available on Github, at (http://tomateba.github.io/p53retriever/).

## Additional files

Additional file 1:
**Complete list of the rules used to attribute the functional score in the pattern search algorithm.**
Additional file 2:
**Complete list of identified REs within human propoter regions (−10 kb, 10 kb from TSS).** The list contains chromosomal coordinates, official gene name, distance from TSS and RE sequence features resulting in the given functional grade. Additional file 3:
**Figures S1–S7, Table S1 and Supplementary Information.**
Additional file 4:
**Comparison between p53retriever classification and lists of published p53 bound regions.**
Additional file 5:
**Gene lists from data curated Ingenuity Pathway (TP53 Canonical Pathway) were compared to prediction and functional ranking of p53 REs.** The RE grade is stated in the name of the various worksheets. A) Grade four and grade five. B) All grade 3, 4, and 5. Additional file 6:
**Complete list of identified REs within ENCODE distal DNAse regions.** The list contains chromosomal coordinates and RE sequence features resulting in the assigned functional grade. Additional file 7:
**qPCR data summarized in Figure** 5 **.** For each gene, time point and treatment time, the average fold change of three biological repeats is presented along with the Standard Deviation. The results obtained with different cell lines are presented in different worksheets

## Abbreviations

RE: Response element
TSS: Transcription start site
3Q: 3/4 binding site
