# Supplementary material for: Whole-genome cartography of p53 response elements ranked on transactivation potential
Source: BMC Genomics. 2015 Jun 17;16(1):464. doi: 10.1186/s12864-015-1643-9 (PMC4470028; doi:10.1186/s12864-015-1643-9)
Supplement: Additional file 3: — Figures S1–S7, Table S1 and Supplementary Information. [file 12864_2015_1643_MOESM3_ESM.pdf]

Whole-genome cartography of p53 response elements ranked on transactivation potential

Toma Tebaldi[1]*, Sara Zaccara[1]*, Federica Alessandrini[1], Alessandra Bisio[1], Yari Ciribilli[1], Alberto Inga[1]

1 – Supplementary Figures

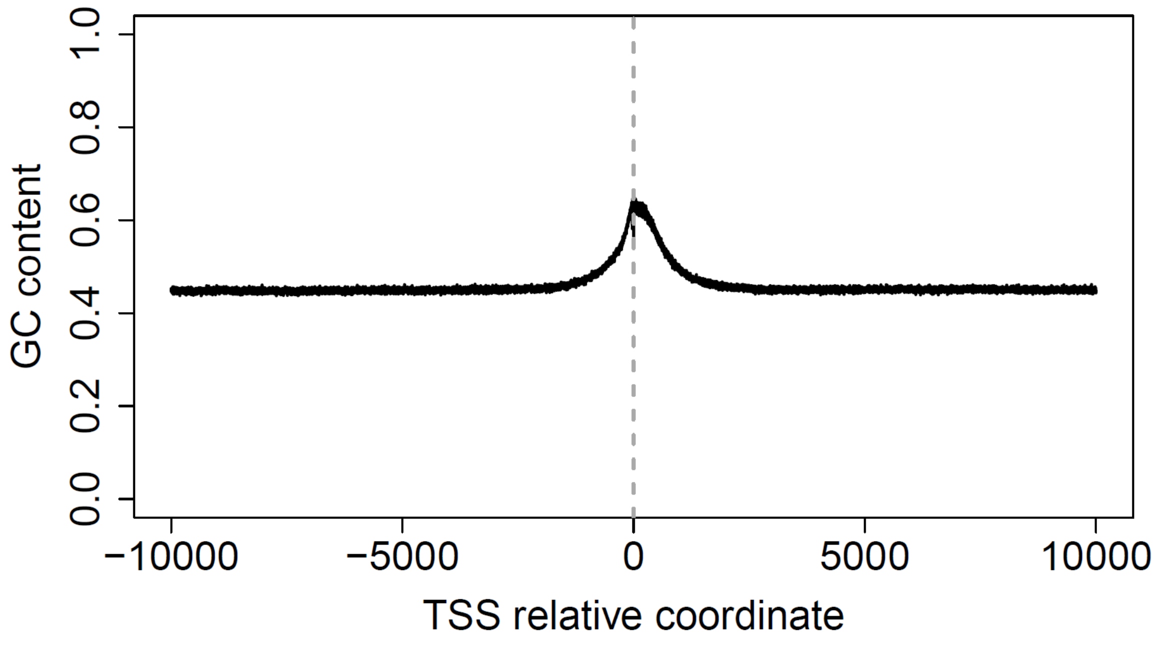

Supplementary Figure 1: positional GC content profile of human genomic regions surrounding all transcriptional start sites (TSS) annotated by UCSC Genome Browser (-10kb, 10kb). The profile is centered on the TSS position.

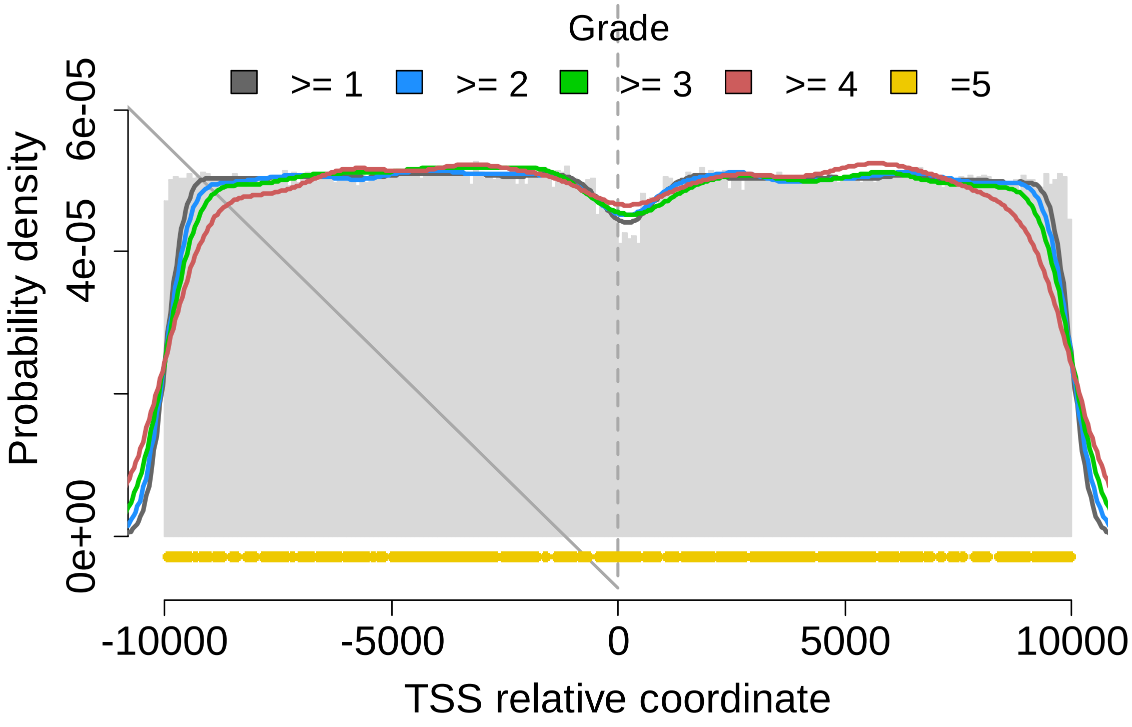

Supplementary Figure 2: p53 random occupancy metaprofile, based on the position of REs found in scrambled human promoter sequences after applying local permutations (to preserve the local GC content). The profile is centered on the TSS position. The grey histogram displays the probability distribution of all REs independently from the grade. Colored lines represent the density distribution of REs with higher grades (the grade threshold corresponding to each color is displayed in the legend). The specific positions of grade 5 REs are dotted in yellow under the histogram.

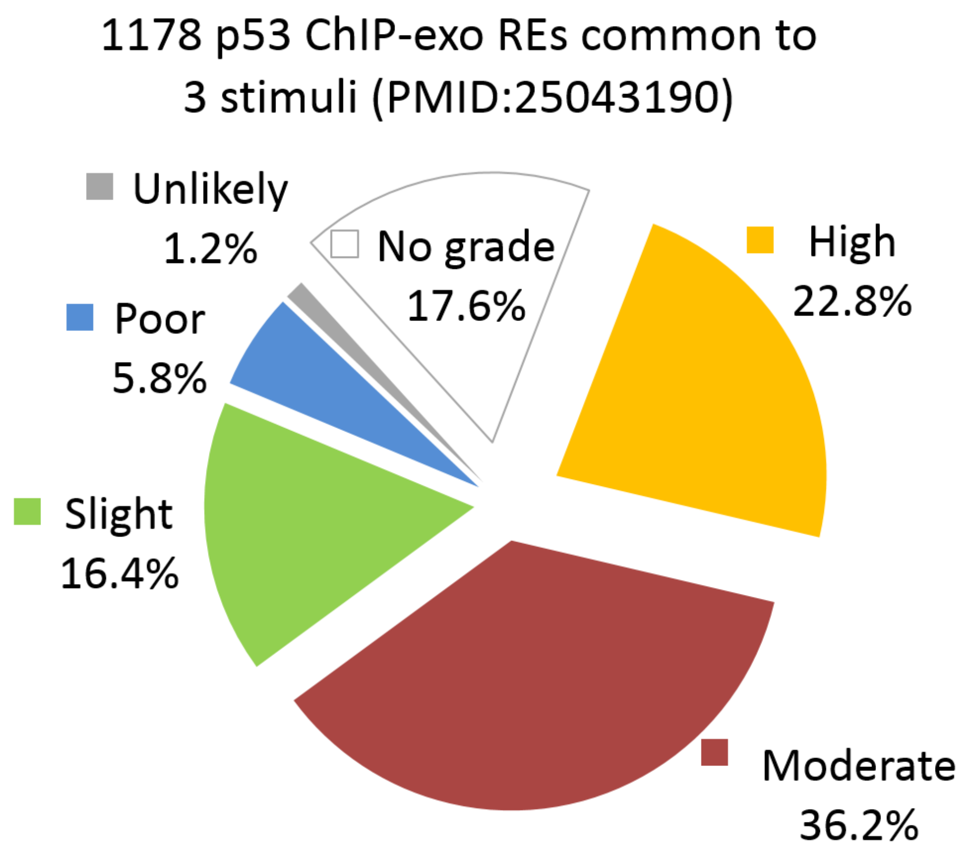

Supplementary Figure 3: pie chart displaying p53retriever classification on a list of 1178 regions consistently identified by ChIP-exo after treatment with Nutlin, Doxorubicin or 5-fluorouracil (PMID:25043190).

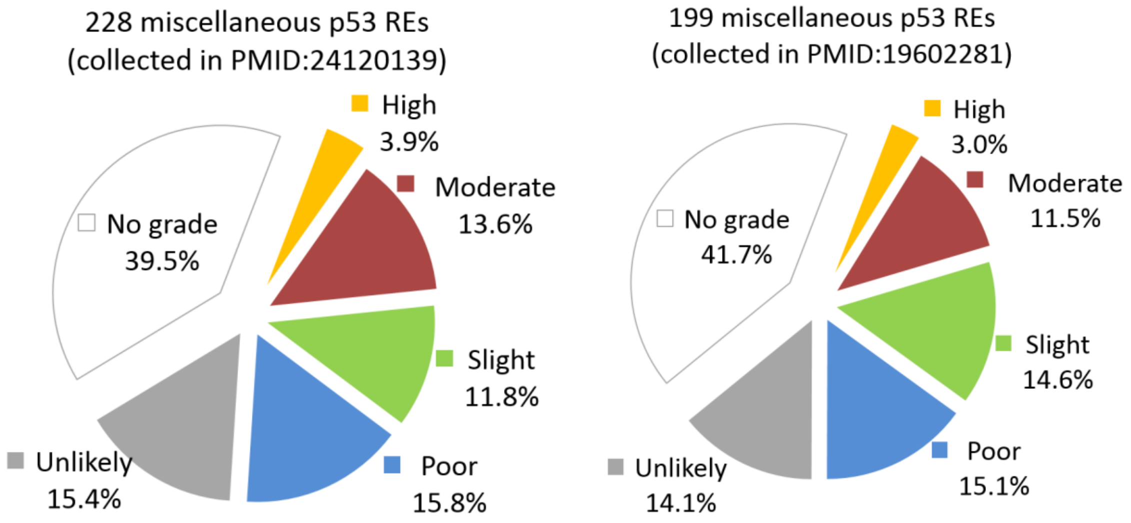

Supplementary Figure 4: Pie chart displaying p53retriever classification on two lists of miscellaneous p53 REs collected in the literature from heterogeneous experimental techniques.

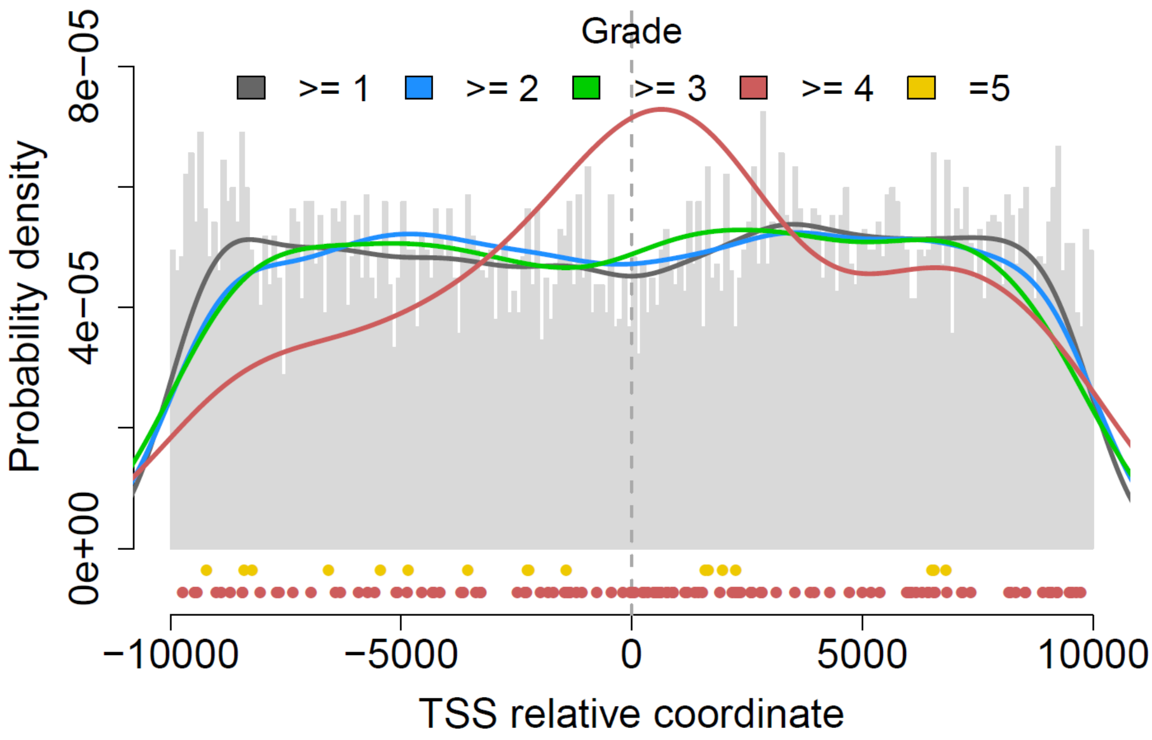

Supplementary Figure 5: p53 occupancy metaprofile, based on the position of REs in human promoters of genes up-regulated upon doxorubicin treatment (PMID:23518503). The profile is centered on the TSS position. The grey histogram displays the probability distribution of all REs independently from the grade. Colored lines represent the density distribution of REs with higher grades (the grade threshold corresponding to each color is displayed in the legend). The specific positions of grade 4 and grade 5 REs are dotted in red and yellow under the histogram.

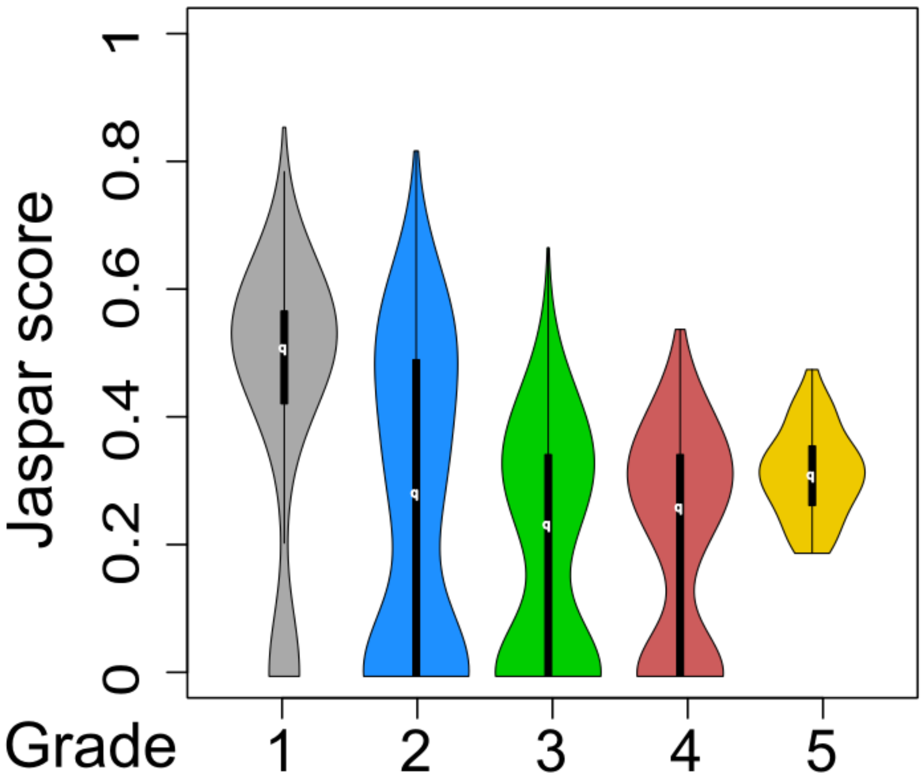

Supplementary Figure 6: Comparison between p53 REs identified in human promoters by p53retriever, and the corresponding score given by Jaspar p53 PWM (MA0106.1), based on SELEX data. REs are divided in 5 groups along the horizontal axis, corresponding to the grade assigned by p53retriever. For each group, the distribution of the scores given by Jaspar PWM is represented as a violin plot, *i.e.* a box plot with a rotated kernel density plot on each side. Jaspar scores range from 0 (the RE is not identified) to 1 (the RE is optimal).

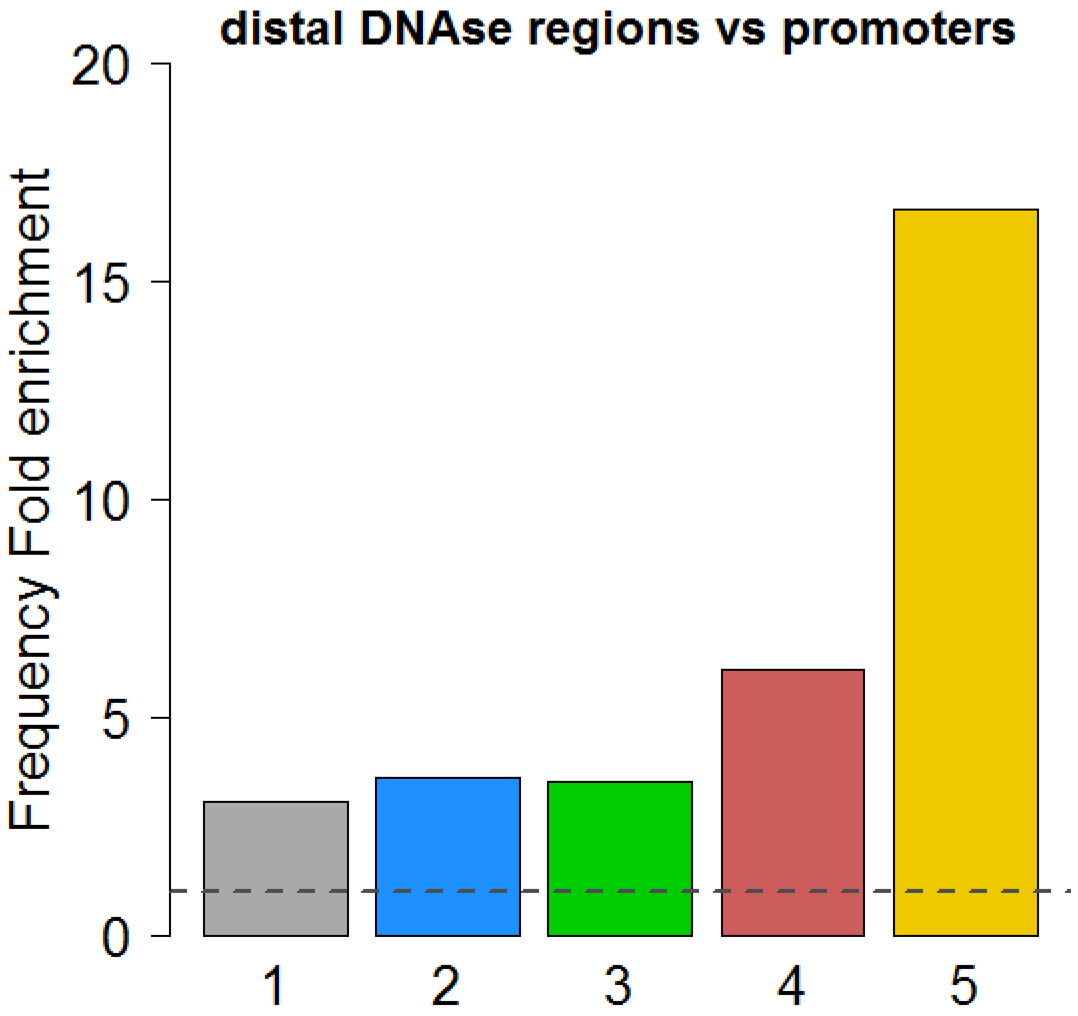

Supplementary Figure 7: Comparison between the frequencies of p53 REs found in human distal DNAse hypersensitive regions, and the frequencies of REs found in promoter sequences. The comparison is shown for each grade. The ratio is 1 if the frequency is the same, > 1 if the frequency is higher in DNAse hypersensitive regions, <1 if the frequency is higher in promoters. The horizontal dotted line indicates the overall ratio between REs, not considering the grade. All enrichments of REs mapped to distal DNAse hypersensitive regions are highly significant (Binomial test p-value < 1E-20).

2 – Supplementary Tables

Human promoters (hg19, -10kb +10kb from TSS)

Score class	Grade	number of REs	Period (nucleotides)
High	5	138	3411739.1
Moderate	4	3148	149561.6
Slight	3	18288	25744.8
Poor	2	52360	8991.9
Unlikely	1	190377	2473.1
Total	All	264311	1781.3

Human distal DNAse hypersensitive regions

Score class	Grade	number of REs	Period (nucleotides)
High	5	144	204730.6
Moderate	4	1198	24608.7
Slight	3	4029	7317.3
Poor	2	11906	2476.2
Unlikely	1	36372	810.5
Total	All	53649	549.5

Random sequences (44% GC)

Score class	Grade	Period mean	Period std dev
High	5	3361344.5	188570.30
Moderate	4	144560.9	1395.65
Slight	3	34370.2	188.57
Poor	2	9773.9	26.97
Unlikely	1	2666.6	4.21
Total	Tot	1946.9	2.48

Scrambled promoters (local permutations)

Score class	Grade	Period mean	Period std dev
High	5	4635598.4	167048.3
Moderate	4	172339.8	1341.1
Slight	3	39480.6	117.6
Poor	2	11109.3	19.1
Unlikely	1	3002.4	3.27
Total	Tot	2200.6	2.19

Supplementary Table 1: statistics on p53 REs identified by p53retriever in the dataset of human promoter sequences, human distal DNAse hypersensitive regions, random DNA sequences and scrambled promoter sequences (with local permutations). The period is the inverse of the frequency, *i.e.* the average distance, expressed in nucleotides, between two REs of the same grade. For random sequences and scrambled promoters, 10 simulations were run: the mean period is reported, with the corresponding standard deviation.

3 - Supplementary Text

Information on nine recently proposed or established direct p53 target genes with mapped functional p53 REs that were confirmed as p53-responsive by qPCR (see Figure 5A).

PDE2A

PDE2A is a cyclic nucleotide (cGMP and cAMP) phosphodiesterase that has been reported as potential marker of cancer treatments [1] and up-regulated in β-catenin mutated adrenocortical carcinomas [2]. Inhibition of PDE2A activity in melanoma cells correlated with G2/M arrest [3]. A PDE2A splice variant was found capable to localize in mitochondria and to regulate respiration [4].

GAS6

GAS6 is a ligand for tyrosine kinase TAM receptors, including AXL. Previously, GAS6 and other genes in a linkage cluster were found overexpressed in aggressive mammary tumors from a p53-null mouse model [5]. Further, high GAS6 expression was an adverse prognostic marker in adult AML patients [6]. High expression of CXCL12 and CXCR4, potentially involved in tumor metastasis [7] was associated with GAS6 expression. However, activated TAM receptors can have both pro- and anti-inflammatory effects, depending on the cell type [8]. As for PDE2A, the physiological role of p53-dependent control of GAS6 expression need to be elucidated.

E2F7

E2F7 is an inhibitory member of the E2F family of transcription factor that does not contain a RB1 binding pocket [9] and can compensate for the loss of RB1 acting by repressing cell cycle progression. p53-dependent activation of E2F7 and a role in controlling proliferation and inducing senescence has been recently reported [10, 11].

APOBEC3H

APOBEC3H belongs to the APOBEC3 family of DNA deaminases, with important role in antiviral defense and also in retro-transposon mobility. APOBEC3C but not 3H was recently discovered as an important mediator of hypermutability in somatic cancer via DNA editing [12]. A role for p53 in the inhibition of retroposition and in counteracting viral infection has been establish and the control of APOBEC3H expression could play an important role in this processes.

KCTD1

KCTD1 is a nuclear protein that functions as a transcriptional repressor. Very recently it was shown to enhance β-catenin degradation, thus suppressing Wnt-mediated signaling [13]. As germline mutations in this gene have been associated with a form of ectodermal dyplasias [14], it would be interesting to assess if KCTD1 can also be a direct target of the p63 transcription factor.

TRIM32

TRIM32 is an E3-ubiquitin ligase protein member of a family that plays a role in HIV infectivity [15], but it has been linked to distinct phenotypes in cancer cells including promotion of apoptosis [16] and differentiation [17].

TGFA

This gene encodes a growth factor that is a ligand for the epidermal growth factor receptor (EGFR), which activates a signaling pathway for cell proliferation, differentiation and development. Moreover, it has been involved in cancer progression. Recent data identified TGFA and other growth factors as major players in the control of anti-apoptotic activities in p53 negative cells. For instance, PDGFRB, IGFR1R and TGFA are upregulated in HCT116 $p53^{-/-}$ cells compared to the HCT116 $p53^{+/+}$ [18].

HRAS

This gene belongs to the Ras oncogene family whose members can bind GTP and GDP, and have intrinsic GTPase activity. These family members are crucial players in many signaling networks that regulate cell-cycle progression, growth, migration, cytoskeletal changes, apoptosis, and senescence [19]. Mutational activation of RAS genes is often present in various tumors in humans, leading to increased invasion & metastasis and decreased apoptosis, due to formation of permanently active GTP-bound form of RAS.

KITLG

KITLG is the ligand for the receptor-type protein-tyrosine kinase KIT. Besides its essential role in the regulation of cell survival and proliferation, the KIT pathway is central to the molecular pathology of different types of cancer [20]. Moreover, mutations of the KIT receptor have been shown to promote tumor formation and the receptor is currently targeted by many therapeutic agents in cancer treatments. More recently, the identification and characterization of the *KITLG* p53-RE SNP supported the hypothesis that polymorphisms in functional p53-binding sites can result in differential p53-dependent transcriptional regulation and cancer risk [21].

4 - Primer sequences

qPCR-primers

GAPDH	Fw	TCCAAAATCAAGTGGGGCGA
	Rv	AGTAGAGGCAGGGATGATGT
B2M	Fw	AGGCTATCCAGCGTACTCCA
	Rv	ATGGATGAAACCCAGACACA
E2F7	Fw	CCCGACTGTCCCTCTTCATCTGCAA
	Rv	AGGCCCGATCCACTTGAAGGCT
GAS6	Fw	CGAGAACGACCCCGAGACGGATT
	Rv	GGTTTTGCACGCAGGTGGCG
PDE2A	Fw	GCGAGCATGGTCCTGGTGCTG
	Rv	AGACAGTTTCCACTCGGGGGAGC
KCTD1	Fw	AGTGCCTCGTCGTGCGTGTG
	Rv	CCCCCACAGGAGCCCACGAT
APOBEC3H	Fw	TGTTACCAGCTGACGCCGCA
	Rv	GGTAGTACAGGCGGGAGGCGA
TRIM32	Fw	GTGGACTCGTCGGAGCCGC
	Rv	TCCCGGAGGGCATCCAGGTT
YAP1	Fw	CCCGACAGGCCAGTACTGATGCAGG
	Rv	TCCTGGGGTCCTGCCATGTTGTT
MAP2K3	Fw	ACCGCCGTCCAGGACCCACT
	Rv	GGCGTGCCGCACCTTCTCTAC
DNAJA1	Fw	CCAATTGCCGAGGTACTGGA
	Rv	CTGGCCATCTTTCATGCCTTT
TGFA	Fw	GGTAGCCGCCTTCCTATTTCC
	Rv	GGACGTGCTGTTCTCCAAGG
KITLG:	F:	GACCTTGTGGAGTGCGTGA
	R:	TTTGGCCTTCCCTTTCTCAGG
HRAS:	Fw	CTGAGGAGCGATGACGGAAT
	Rv	CCCATCAATGACCACCTGCT

ChIP-primers

DICER:	Fw	TGTTGCTCTTGTCTGAACTGC
	Rv	AGGGTAAGCCTTGGTATCCT
YAP1:	Fw	GACAGGAAACTGGGGGTTCA
	Rv	TGCACAAGCCCATTCTAAGGA
MAP2K3	Fw	GACCCTGCTTGTCTGGATGT
	Rv	GACCACATTGTCACGAAGCC
DNAJA1	Fw	GGCGTATATGGGAAACTGCG
	Rv	TGGACAAAGCACAGGGGAA

5 – Supplementary References

1. Frolov A, Chahwan S, Ochs M, Arnoletti JP, Pan ZZ, Favorova O, Fletcher J, von Mehren M, Eisenberg B, Godwin AK: **Response markers and the molecular mechanisms of action of Gleevec in gastrointestinal stromal tumors**. *Mol Cancer Ther* 2003, **2**(8):699-709.
2. Durand J, Lampron A, Mazzuco TL, Chapman A, Bourdeau I: **Characterization of differential gene expression in adrenocortical tumors harboring beta-catenin (CTNNB1) mutations**. *The Journal of clinical endocrinology and metabolism* 2011, **96**(7):E1206-1211.
3. Morita H, Murata T, Shimizu K, Okumura K, Inui M, Tagawa T: **Characterization of phosphodiesterase 2A in human malignant melanoma PMP cells**. *Oncol Rep* 2013, **29**(4):1275-1284.
4. Acin-Perez R, Russwurm M, Gunnewig K, Gertz M, Zoidl G, Ramos L, Buck J, Levin LR, Rassow J, Manfredi G *et al*: **A phosphodiesterase 2A isoform localized to mitochondria regulates respiration**. *J Biol Chem* 2011, **286**(35):30423-30432.
5. Abba MC, Fabris VT, Hu Y, Kittrell FS, Cai WW, Donehower LA, Sahin A, Medina D, Aldaz CM: **Identification of novel amplification gene targets in mouse and human breast cancer at a syntenic cluster mapping to mouse ch8A1 and human ch13q34**. *Cancer Res* 2007, **67**(9):4104-4112.
6. Whitman SP, Kohlschmidt J, Maharry K, Volinia S, Mrozek K, Nicolet D, Schwind S, Becker H, Metzeler KH, Mendler JH *et al*: **GAS6 expression identifies high-risk adult AML patients: potential implications for therapy**. *Leukemia* 2014, **28**(6):1252-1258.
7. Acharyya S, Oskarsson T, Vanharanta S, Malladi S, Kim J, Morris PG, Manova-Todorova K, Leversha M, Hogg N, Seshan VE *et al*: **A CXCL1 paracrine network links cancer chemoresistance and metastasis**. *Cell* 2012, **150**(1):165-178.
8. van der Meer JH, van der Poll T, van 't Veer C: **TAM receptors, Gas6, and protein S: roles in inflammation and hemostasis**. *Blood* 2014, **123**(16):2460-2469.
9. Chen HZ, Tsai SY, Leone G: **Emerging roles of E2Fs in cancer: an exit from cell cycle control.** *Nat Rev Cancer* 2009, **9**(11):785-797.
10. Aksoy O, Chicas A, Zeng T, Zhao Z, McCurrach M, Wang X, Lowe SW: **The atypical E2F family member E2F7 couples the p53 and RB pathways during cellular senescence.** *Genes Dev* 2012, **26**(14):1546-1557.
11. Carvajal LA, Hamard PJ, Tonnessen C, Manfredi JJ: **E2F7, a novel target, is up-regulated by p53 and mediates DNA damage-dependent transcriptional repression**. *Genes Dev* 2012, **26**(14):1533-1545.
12. Roberts SA, Lawrence MS, Klimczak LJ, Grimm SA, Fargo D, Stojanov P, Kiezun A, Kryukov GV, Carter SL, Saksena G *et al*: **An APOBEC cytidine**

deaminase mutagenesis pattern is widespread in human cancers. *Nat Genet* 2013, **45**(9):970-976.

13. Li X, Chen C, Wang F, Huang W, Liang Z, Xiao Y, Wei K, Wan Z, Hu X, Xiang S *et al*: **KCTD1 suppresses canonical Wnt signaling pathway by enhancing beta-catenin degradation**. *PLoS One* 2014, **9**(4):e94343.
14. Marneros AG, Beck AE, Turner EH, McMillin MJ, Edwards MJ, Field M, de Macena Sobreira NL, Perez AB, Fortes JA, Lampe AK *et al*: **Mutations in KCTD1 cause scalp-ear-nipple syndrome**. *Am J Hum Genet* 2013, **92**(4):621-626.
15. Uchil PD, Quinlan BD, Chan WT, Luna JM, Mothes W: **TRIM E3 ligases interfere with early and late stages of the retroviral life cycle**. *PLoS pathogens* 2008, **4**(2):e16.
16. Ryu YS, Lee Y, Lee KW, Hwang CY, Maeng JS, Kim JH, Seo YS, You KH, Song B, Kwon KS: **TRIM32 protein sensitizes cells to tumor necrosis factor (TNFalpha)-induced apoptosis via its RING domain-dependent E3 ligase activity against X-linked inhibitor of apoptosis (XIAP)**. *J Biol Chem* 2011, **286**(29):25729-25738.
17. Sato T, Okumura F, Iguchi A, Ariga T, Hatakeyama S: **TRIM32 promotes retinoic acid receptor alpha-mediated differentiation in human promyelogenous leukemic cell line HL60**. *Biochem Biophys Res Commun* 2012, **417**(1):594-600.
18. Tian K, Rajendran R, Doddananjaiah M, Krstic-Demonacos M, Schwartz JM: **Dynamics of DNA damage induced pathways to cancer**. *PLoS One* 2013, **8**(9):e72303.
19. Milinkovic VP, Skender Gazibara MK, Manojlovic Gacic EM, Gazibara TM, Tanic NT: **The impact of TP53 and RAS mutations on cerebellar glioblastomas**. *Experimental and molecular pathology* 2014, **97**(2):202-207.
20. Gilbert D, Rapley E, Shipley J: **Testicular germ cell tumours: predisposition genes and the male germ cell niche**. *Nat Rev Cancer* 2011, **11**(4):278-288.
21. Zeron-Medina J, Wang X, Repapi E, Campbell MR, Su D, Castro-Giner F, Davies B, Peterse EF, Sacilotto N, Walker GJ *et al*: **A polymorphic p53 response element in KIT ligand influences cancer risk and has undergone natural selection**. *Cell* 2013, **155**(2):410-422.
